# Dual inflow without circulatory arrest for hemiarch replacement

**DOI:** 10.1186/s13019-018-0826-2

**Published:** 2019-01-10

**Authors:** Tae Yun Kim, Kyung Hwa Kim

**Affiliations:** 1 Department of Thoracic and Cardiovascular Surgery, Chonbuk, National University Medical School, Chonbuk National University Hospital, 20 Geonji-Ro, Geumam-dong, Deokjin-gu, Jeonju, 54907 South Korea; 2 Research Institute of Clinical Medicine of Chonbuk National University and Biomedical Research Institute of Chonbuk National University Hospital, 20 Geonji-Ro, Geumam-dong, Deokjin-gu, Jeonju, 54907 South Korea

**Keywords:** Dual inflow, Hemiarch replacement, No circulatory arrest

## Abstract

**Background:**

Open distal graft to proximal aortic arch anastomosis is central to a hemiarch replacement. Even if the ischemic tolerance time of several organs during circulatory arrest (CA) at normothermia is much longer than that of the brain, very little is known about the safety and clinical efficacy of moderate hypothermia for organ protection during the average duration of CA needed for aortic arch replacement. Hemiarch replacement using the standard techniques of deep hypothermia and antegrade perfusion has often ignored the effects of prolonged distal body CA. Maintenance of distal organ, especially the liver and kidney, perfusion reduces the risk of postoperative renal dysfunction and coagulopathy.

**Case presentation:**

A 72-year-old female patient was referred to our institute due to chest discomfort. Radiologic investigations revealed a giant aneurysm of the ascending aorta extending but confined to the proximal aortic arch. We performed an alternative technique for hemiarch replacement using a dual inflow source.

**Conclusions:**

Although this technique cannot apply to all aneurysmal aortic diseases, our basic technique involving the use of dual inflow may be well suited for standard hemiarch replacement that is confined to the proximal aortic arch, given the shortening of the bypass and ischemic times.

## Background

Although the use of selective antegrade cerebral perfusion (SACP) during circulatory arrest (CA) has allowed aortic arch repair to be performed safely with moderate hypothermic CA [1], the susceptibility of the distal organ to ischemia often has been ignored during CA [2]. In particular, visceral organ injury cannot be guaranteed during moderate hypothermic CA when cerebral perfusion is used alone. We present an alternative technique for hemiarch replacement using a dual inflow strategy that does not require deep hypothermic CA.

## Case presentation

**Fig. 1:**
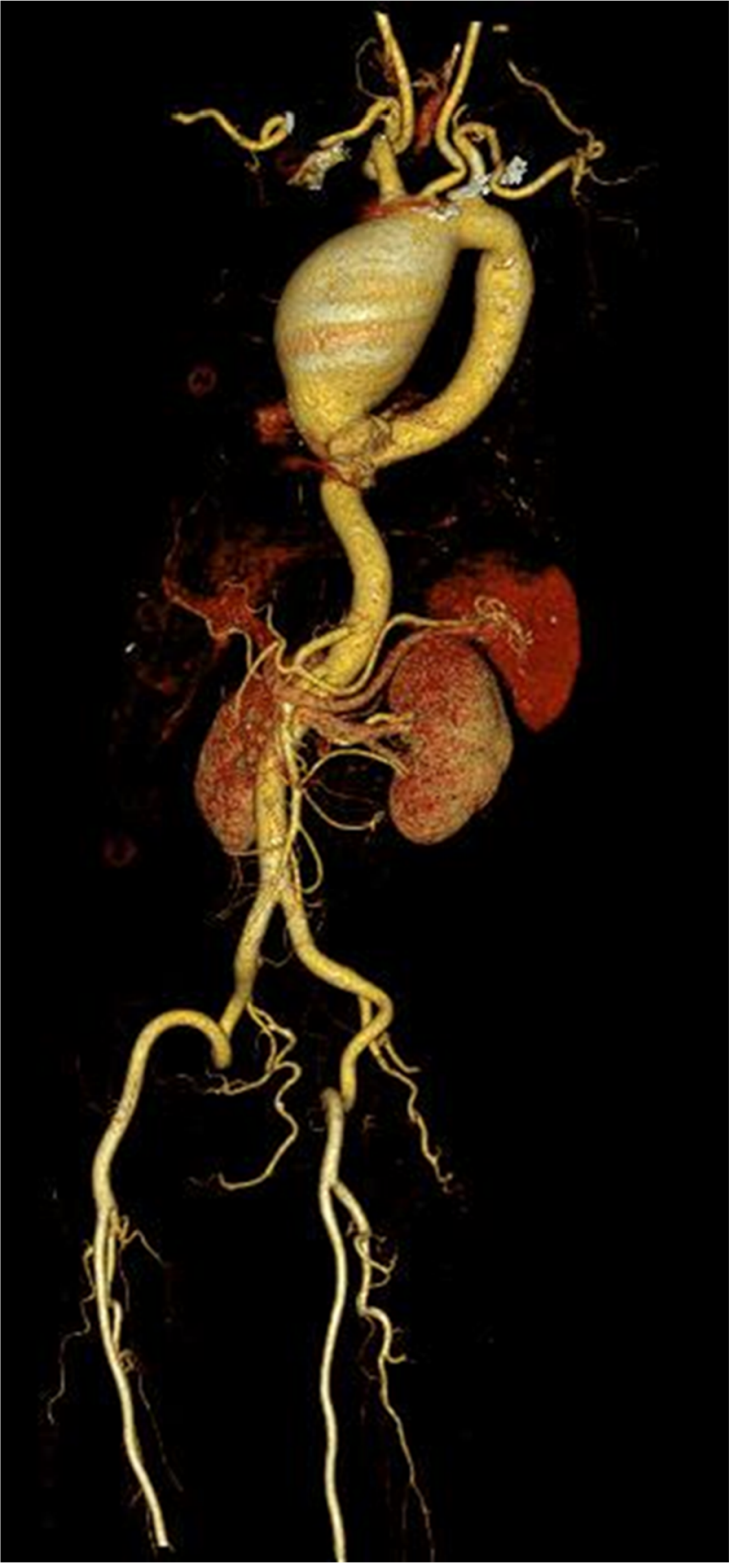
Computed tomography showing a giant ascending aortic aneurysm extending but confined to the proximal aortic arch

A 72-year-old female patient was referred to our institute due to chest discomfort. The radiologic investigations revealed a giant aneurysm of the ascending aorta, extending but confined to the proximal aortic arch and 7.5 cm in size (Fig. 1).

**Fig. 2:**
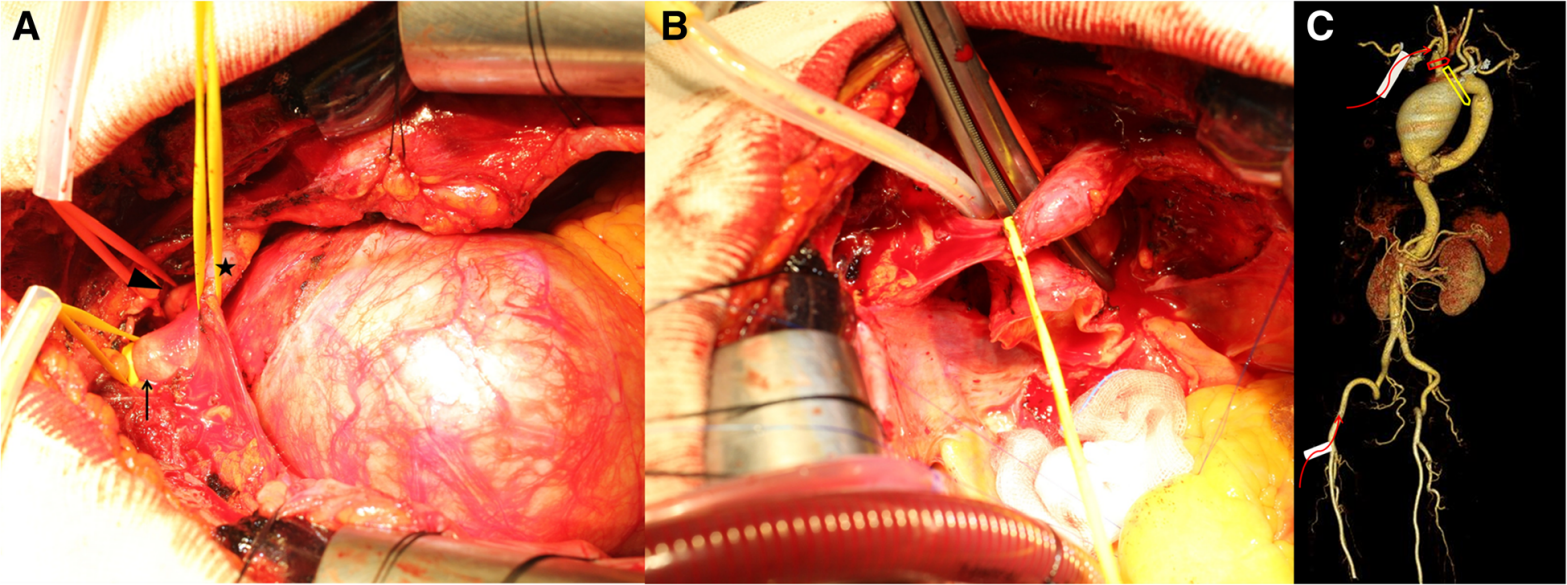
Intra-operative images. **a** Vessel loops with tourniquets were placed around the completely freed innominate artery (arrow), left common carotid artery (arrowhead) and innominate vein (asterisk). **b** The applied mid-arch aorta clamp between the innominate artery and the left common carotid artery. **c** The schematic diagram of the dual inflow technique; the vascular graft of right axillary and right femoral artery cannulation for the dual inflow source (white box and curved red arrow) with right innominate artery isolation (red square) and applied mid-arch aorta clamp (yellow square)

**Fig. 3:**
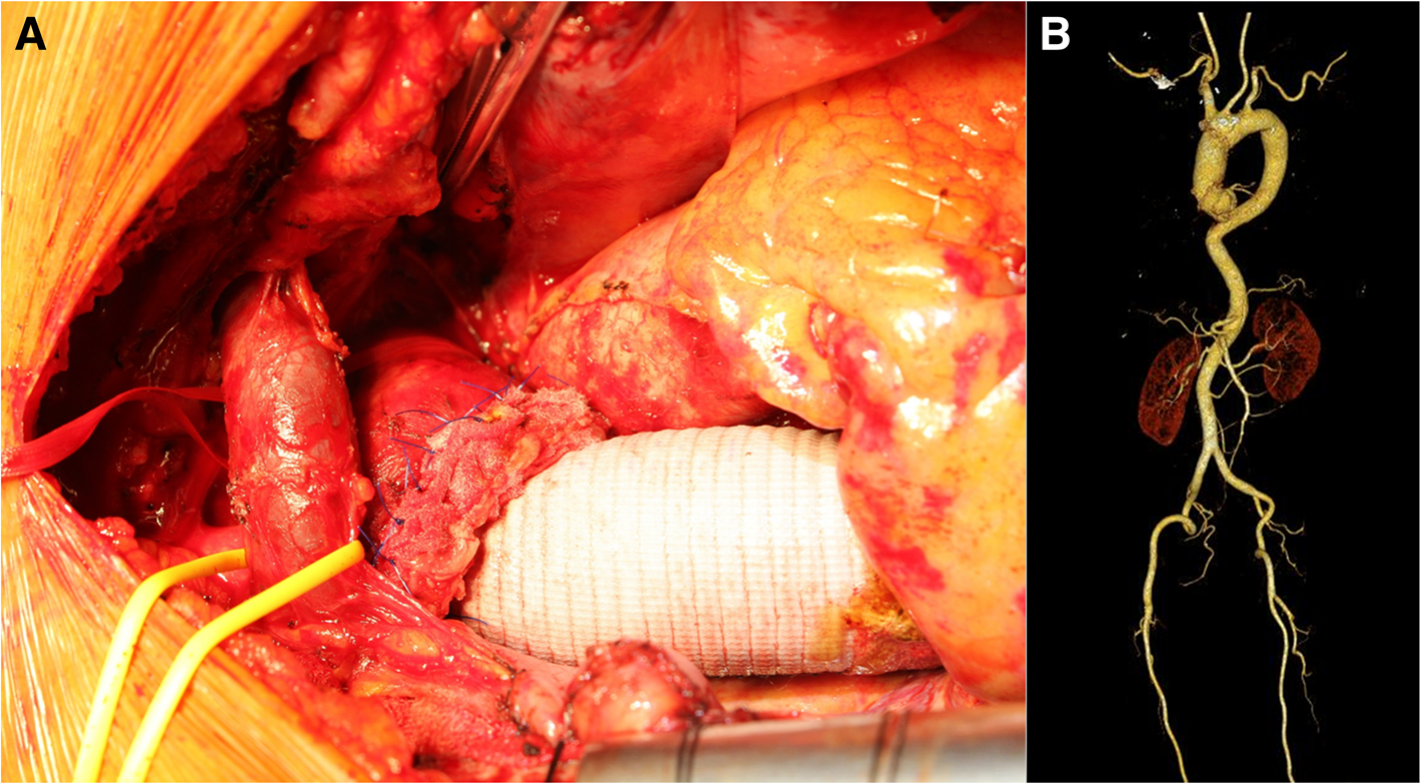
**a** Intra-operative image of the completion of the hemiarch aorta replacement. **b** Follow-up computed tomography showing a well-reconstructed aorta

We performed a median sternotomy with initial arterial cannulation of the right axillary artery using an 8 mm vascular graft and the right femoral artery for a second arterial line with a Y-limb preparing the circuit. The arm of the circuit going to the femoral artery was clamped. A two-stage venous cannula was inserted into the right atrium. The bypass was initiated via the right axillary arterial line and systemic cooling was applied to reach a bladder temperature of 32 °C. During cooling, vessel loops with tourniquets were placed around the completely freed innominate and left common carotid arteries (Fig. 2a). Once the vessel loop on the innominate artery was secured, a cross clamp was applied distal to the innominate artery in an oblique fashion (Fig. 2b), and then the arm of the circuit going to the femoral artery was unclamped. One pump circuit was used for the axillary and femoral artery, and the perfusion pressure was maintained at approximately 50~60 mmHg, as measured in the bilateral radial artery. Cerebral saturation was monitored using near-infrared spectroscopy (NIRO 300: Hamamatsu Phototonics, Hamamatsu, Japan). The anterior arch resection was performed via a long beveled incision, starting at the right side of the origin of the innominate artery and ending in the lesser curvature of the arch in front of the clamp. The distal hemiarch anastomosis was carried out by means of a continuous 3–0 polypropylene suture with interrupted U-shape pledget stitches, and the graft was cross-clamped proximally; the pump flow was then returned to the antegrade arterial inflow through the axillary arterial line, and the proximal end of the aortic graft was completed just up to the sinotubular junction (Fig. 3a). The postoperative course was uneventful. Postoperative computed tomography showed a well-reconstructed aorta (Fig. 3b). At the one-year follow-up, there was no sign of a thromboembolic event or renal or hepatic failure.

## Discussion and conclusions

Open distal graft to proximal aortic arch anastomosis is the sine qua non of hemiarch aortic replacement. Many institutions have moved to a strategy of mild to moderate hypothermia with SACP for aortic arch replacement [1, 3]. Even if several organs, such as the liver, the kidney or even the spinal cord, have a much longer ischemic tolerance time at normothermia during CA than the brain, very little is known about the safety and clinical efficacy of mild-to-moderate hypothermia for organ protection during the average CA time needed for aortic arch replacement. Even short periods of cerebral CA and arch branch manipulation have been shown to be deleterious for higher mental function and to create an opportunity for cerebral embolization of air and debris [1, 2]. Using unilateral cerebral perfusion, often via the right axillary artery, while seeming to avert cerebral CA, still poses issues related to contralateral hemispheric hypoperfusion or ipsilateral hyperperfusion [2]. Perioperative hepatic and renal dysfunction substantially contribute to postoperative morbidity and mortality [2]. Arch replacement using standard techniques of deep hypothermia and antegrade perfusion often ignore the effects of prolonged distal body CA. Maintenance of distal organ and especially liver and kidney perfusion reduces the risk of postoperative renal dysfunction and coagulopathy [2]. Thus, our proposed technique can avoid many of the shortcomings of standard arch replacement strategies. A dual inflow source is important not only for providing distal body perfusion during arch branch reconstruction but also for lowering perfusion pressure gradients, thereby avoiding problems such as hyperperfusion of the right hemisphere that arise when right axillary cannulation is used alone [2]. The bypass time for the patient was 60 min.

Our proposed technique can be applied safely, but there are some cautions. Care must be taken to avoid exposure to the recurrent laryngeal nerve during arch vessel dissection or clamping the mid-arch, as the technique requires adequate and precise exposure of the aortic arch.

Although this technique cannot apply to all aneurysmal aortic diseases, our basic technique using dual inflow may be well suited for standard hemiarch replacement cases in which the aneurysm is confined to the proximal aortic arch, given the shortening of the bypass and ischemic times.

## Abbreviations

CA: Circulatory arrest abbreviations
SACP: Selective antegrade cerebral perfusion
